# Robust ROI Detection in Whole Slide Images Guided by Pathologists’ Viewing Patterns

**DOI:** 10.1007/s10278-024-01202-x

**Published:** 2024-08-09

**Authors:** Fatemeh Ghezloo, Oliver H. Chang, Stevan R. Knezevich, Kristin C. Shaw, Kia Gianni Thigpen, Lisa M. Reisch, Linda G. Shapiro, Joann G. Elmore

**Affiliations:** 1 Paul G. Allen School of Computer Science and Engineering, University of Washington, Seattle, WA USA; 2 Department of Laboratory Medicine and Pathology, University of Washington, Seattle, WA USA; 3 Pathology Associates, Clovis, CA USA; 4 VA Medical Center, Portland, OR USA; 5 Department of Medicine, University of Washington, Seattle, WA USA; 6 Department of Biostatistics, University of Washington, Seattle, WA USA; 7 Department of Medicine, David Geffen School of Medicine, University of California, Los AngelesLos Angeles, CA USA

**Keywords:** Digital pathology, Medical image analysis, Deep learning, Region of interest, Saliency detection, Image reconstruction

## Abstract

Deep learning techniques offer improvements in computer-aided diagnosis systems. However, acquiring image domain annotations is challenging due to the knowledge and commitment required of expert pathologists. Pathologists often identify regions in whole slide images with diagnostic relevance rather than examining the entire slide, with a positive correlation between the time spent on these critical image regions and diagnostic accuracy. In this paper, a heatmap is generated to represent pathologists’ viewing patterns during diagnosis and used to guide a deep learning architecture during training. The proposed system outperforms traditional approaches based on color and texture image characteristics, integrating pathologists’ domain expertise to enhance region of interest detection without needing individual case annotations. Evaluating our best model, a U-Net model with a pre-trained ResNet-18 encoder, on a skin biopsy whole slide image dataset for melanoma diagnosis, shows its potential in detecting regions of interest, surpassing conventional methods with an increase of 20%, 11%, 22%, and 12% in precision, recall, F1-score, and Intersection over Union, respectively. In a clinical evaluation, three dermatopathologists agreed on the model’s effectiveness in replicating pathologists’ diagnostic viewing behavior and accurately identifying critical regions. Finally, our study demonstrates that incorporating heatmaps as supplementary signals can enhance the performance of computer-aided diagnosis systems. Without the availability of eye tracking data, identifying precise focus areas is challenging, but our approach shows promise in assisting pathologists in improving diagnostic accuracy and efficiency, streamlining annotation processes, and aiding the training of new pathologists.

## Introduction

Cutaneous melanoma, the most lethal form of skin cancer, most commonly originates from melanocytes at the dermal–epidermal junction. The global prevalence of melanoma is on the rise, establishing it as one of the most commonly diagnosed cancers in adults [1, 2]. Early detection and effective management are paramount due to its high mortality rate upon metastasis [3]. The diagnosis of melanoma requires a skin biopsy followed by a meticulous examination by a pathologist. However, histopathological analysis of biopsy specimens can be subjective and susceptible to diagnostic errors [4]. In the medical field, diagnostic errors contribute to 10% of patient deaths and constitute a leading source of medical malpractice claims [5]. The complex nature of melanoma diagnosis arises from its diverse presentations in terms of size, morphology, and growth patterns [3]. Pathologists are tasked with identifying specific image regions displaying pathological characteristics, relying on their clinical expertise to interpret these visual cues and either confirm or exclude a particular diagnosis [6].

Over the past decades, advances in technology coupled with the growing adoption of machine learning techniques have profoundly reshaped medical care, especially with its integration into healthcare systems [7–14]. With the advent of whole slide imaging, the entire glass slides can be digitized into high-resolution images, allowing pathologists to conveniently view and analyze tissue samples on a computer [15, 16]. Devices such as eye tracking and viewport tracking, where a viewport is the visible rectangular area of the image on a pathologist’s computer screen, allow us to record how pathologists interact with the information on digital whole slide images. Incorporating tracking devices into this process allows researchers to better understand pathologists’ interpretive behavior and interaction with digital slides [17–19]. This has transformed the histopathology field by gaining an understanding of the diagnostic decision-making process.

Detecting regions of interest (ROIs) on a whole slide image (WSI) involves a visual assessment of an image to locate regions with the most relevant and representative pathology. An eye tracking study highlights the crucial role of fixating on a consensus-defined ROI, as failure to do so can lead to the pathologist overlooking these critical areas [20]. Previous studies show a connection between pathologists’ viewing behaviors and diagnostic accuracy [21, 22]. This study hypothesizes that computer-aided diagnosis (CAD) systems might benefit from incorporating viewing behavior data. Hence, automatic ROI recognition is a reasonable first step to developing an automated diagnosis system. Marzahl et al. show that automatic annotations on microscopy slides increased consensus among experts and increased accuracy in deep learning classifiers more than manual annotations, ensuring more consistent and repeatable results which is highly desirable in the medical field [23].

Previous ROI detection systems have been developed in different frameworks including object detection [24–29], tissue segmentation [30–34], classification [35], CNN-based feature extraction [36–38], and content-based histopathology image retrieval [39–41]. These methods mostly rely on pathologists’ manual ROI annotations, which are costly, time-intensive, and require domain expertise. However, pathologists’ viewing behavior data collected during their routine diagnosis sessions on digital viewers offers a rich and efficient source of information for ROI detection [42]. While Mercan et al. employed pathologists’ viewport tracking for breast biopsy images [35] and Zou et al. used ophthalmologists’ eye tracking for retinal images to localize diabetic macular edema ROIs [43], these models are restricted by their reliance on basic image attributes like color and texture. These models face challenges in generalization and performance across varied conditions such as different scanners, color distributions, and image types. Moreover, research in computer vision has demonstrated that deep learning algorithms can outperform algorithms that use hand-crafted features [44, 45].

This paper proposes an innovative method combining information on pathologists’ viewing behavior and deep image features to generate heatmaps indicating diagnostically relevant areas on WSI. A heatmap is a visual representation of data where varying colors highlight the significance or frequency of pathologists’ attention on specific regions. These heatmaps guide our model, enabling the reconstruction of heatmaps for input images. Our approach integrates pathologists’ domain knowledge with deep image features, enabling robust ROI detection. The model’s effectiveness is demonstrated by evaluating its performance on WSIs of skin biopsies of melanocytic lesions. The proposed model excels by utilizing pathologists’ viewing behaviors, offering the potential to assist pathologists in clinical training programs, clinical practices, and the development of CAD systems. The key contributions of our study include:

- A novel system that emphasizes viewing behaviors for ROI detection,
- Broad applicability to varied pathology types,
- High recall in ROI identification,
- performance improvement of computer-aided diagnosis models by incorporating ROI detection result as supplementary signals.

## Materials and Methods

This section provides an overview of our dataset, including its characteristics and statistics. We outline the steps taken to process the viewport data, extract ROIs from pathologists’ viewing behavior, and generate heatmaps. Additionally, we explain how these heatmaps are integrated into our ROI detection pipeline. Moreover, we discuss the evaluation methodology employed to assess our model’s performance in predicting heatmaps of clinically important regions.

### Dataset and Pre-processing

In this section, we provide an in-depth overview of our dataset and the related pre-processing methods. We start by introducing the skin biopsy WSIs dataset. Further details will be provided on the pathologists’ viewport data and its collection methodology. Next, we will define our measure of diagnostic accuracy, which is based on a consensus reference diagnosis. Concluding this section, we describe how we selected and split our data for the study.

#### Skin Biopsy WSIs

The skin biopsy WSIs in this study are from the prior M-Path study [4, 46] in which skin biopsy specimens of melanocytic lesions (*N* = 240) were randomly selected from available stored specimens at Dermatopathology Northwest in Bellevue, Washington. Data used in the current study was collected and de-identified prior to this study; thus, the current study does not involve any sensitive patient health information. The hematoxylin and eosin (H&E) stained slides were selected with stratification based on the patient’s age and the original diagnosis. Each glass slide was scanned at 40 × magnification using a Hamamatsu NanoZoomer 2.0-RS digital slide scanner to generate digital WSIs. These cases were classified into 5 diagnostic classes using the original MPATH-Dx scheme [47]. The number of biopsy cases in each class and example diagnostic terms for each class are as follows: 25 cases in class 1 (nevus/mild atypia), 36 cases in class 2 (moderate atypia/dysplasia), 60 cases in class 3 (severe dysplasia/melanoma in situ), 58 cases in class 4 (stage pT1a invasive melanoma), and 61 cases in class 5 (stage pT1b or higher invasive melanoma). The details of the dataset collection and classification can be found elsewhere [4, 46]. To be consistent with the latest revision of the MPATH-Dx classification scheme [48], we combined classes 1 and 2 in the original dataset. This leaves us with a more balanced data distribution among four different classes. Table 1 summarizes our dataset distribution among the four MPATH-Dx classes. Due to stringent privacy considerations, ethical constraints, and institutional policies, our dataset is not publicly available for general release. However, interested individuals can contact authors for more information.

#### Pathologists’ Viewport Data

Pathologists’ viewport data from the prior M-Path study [49] was collected using an online digital slide viewer that was developed using HD View SL, Microsoft’s open-source Silverlight gigapixel picture viewer. The viewer allowed pathologists to pan around the image and zoom in and out up to × 60 magnification. The web-based viewer automatically logged the viewport tracking data as pathologists viewed each slide. A viewport is a rectangular area of the image that is visible on the pathologist’s computer screen at any time during their interpretation. For each interpretation (pair of pathologist and case), a list of viewport coordinates, magnification (zoom) level, and time stamps were recorded.

This de-identified dataset includes viewport tracking data from two groups of pathologists: community pathologists and M-Path consensus reference panel. Community pathologists who were recruited for the M-Path study had completed residency and/or dermatopathology post-doctoral training, had interpreted skin specimens in their clinical practices in the preceding year, and planned to do so for the next two years. Three dermatopathologists participated in this study as members of the M-Path consensus panel, each with expertise in cutaneous melanocytic lesions (see “Consensus Reference Diagnosis and Relationship to Diagnostic Accuracy” section). Each of the pathologists from these two groups viewed and diagnosed these cases independently, and their viewport logs are available. Each case in our dataset was interpreted by one consensus reference panel dermatopathologist and an average of five community pathologists.

#### Consensus Reference Diagnosis and Relationship to Diagnostic Accuracy

The consensus reference panel of three dermatopathologists with internationally recognized expertise independently interpreted the full set of 240 cases in glass slide format and then participated in a series of six full-day review meetings as part of the earlier M-Path study [46]. Utilizing a multi-headed microscope during the review meetings, they agreed on a consensus diagnosis for each case using a modified Delphi approach [46] and wrote case guidelines together for each of the 240 cases. Cases were then digitized, and an additional dermatopathologist from the M-Path research team joined the panel to determine a consensus rectangular region as the ROI for each case. ROIs were selected by the expert dermatopathologists as the area that best supported their diagnosis and best represented the critical features on the slide, as described in the aforementioned case guidelines. These variable-sized ROIs provide valuable, diagnostically important information, and can be extracted using their coordinates. In this project, we evaluate diagnostic accuracy by assessing the agreement between the diagnosis provided by community pathologists and the consensus diagnosis determined by our panel of three internationally recognized dermatopathologists. Diagnostic error is a metric used to measure the divergence between a pathologist’s diagnosis and the consensus diagnosis. For instance, if the consensus diagnosis is class 3 and the pathologist’s diagnosis is class 2 or class 4, it would be considered a 1-class error. Note that these are the diagnostic accuracy and error of the pathologists and are unrelated to the accuracy of the proposed method.

#### Data Split

From the M-Path dataset, which contained 240 patients’ digital WSIs of their skin biopsies, we narrowed down our selection to 172 cases. This selection was based on the availability of viewport tracking data for a case and the inclusion of interpretations (pathologist, case) with a maximum of 1-class error, as defined in the “Consensus Reference Diagnosis and Relationship to Diagnostic Accuracy” section. As a consequence of this criterion, a total of 856 interpretations (an average of 5 pathologists independently interpreted each case) were retained out of the initial 1036 interpretations. We analyze our WSIs at 10 × magnification as they provide enough clinical information to allow diagnostic classification by the pathologists for most cases, yet are of reasonable size for processing. To address the challenges posed by the large size and variability of WSIs, various processing techniques can be applied. While one approach involves down-sampling and resizing the WSIs to a fixed size, this can lead to a loss of valuable information. Instead, we employ a cropping strategy, dividing the WSIs into non-overlapping patches of size 256 × 256 and 512 × 512. By processing each patch individually, we can retain important details while effectively managing the computational requirements associated with the analysis of WSIs. Our dataset was split and stratified based on the consensus MPATH-Dx class of each case to train (60%), validation (20%), and test (20%) sets. This ensures that each subset contains a representative distribution of the four different MPATH-Dx classes. In Table 1, we provide a summary of the size of the train, validation, and test subsets.

**Table 1:** Dataset summary

MPATH-Dx Class	# of Cases (Train 60%)	# of Cases (Validation 20%)	# of Cases (Test 20%)	Total
1 and 2	26	9	9	44
3	26	9	9	44
4	24	8	8	40
5	26	9	9	44
Total cases	102	35	35	172
Total interpretations	507	180	169	856
Total patches (256 × 256)	96614	15812	23440	135866
Total patches (512 × 512)	26699	4691	6604	37994

### Methods

In this section, we outline the various components of our pipeline. Initially, we detail the method of extracting important regions from the viewport data. Following that, we delve into the process of generating heatmaps based on these critical viewports. Furthermore, we present the network architecture and discuss the evaluation metrics employed in our study. Our codebase is available at: https://github.com/fGhezloo/ROI-Localization-melanoma.

#### Extracting Viewing ROIs

We employed the method proposed by Mercan et al. [35] to extract diagnostically important areas from WSIs based on pathologists’ viewing behavior. This method involves three behaviors: zoom peaks, slow pannings, and fixations. We describe these three behaviors below and more details about their methodology can be found elsewhere [35].

- **Zoom peaks:** These are log entries where the zoom level is higher than the previous and the next entries. A zoom peak identifies a region where the pathologist intentionally zoomed to look at a higher magnification.
- **Slow pannings:** These are the log entries where the zoom level remains constant, and the displacement between the center of two viewports is small (less than 100 pixels). Slow pannings are intended for investigating a slightly larger area without completely moving the viewport.
- **Fixations:** These are the log entries where the duration is longer than 2 s. Fixations identify the areas to which a pathologist focuses extra attention by looking at them longer. Entries longer than 1 min were excluded due to the assumption that the pathologist was not actively interpreting during that time.

**Fig. 1:**
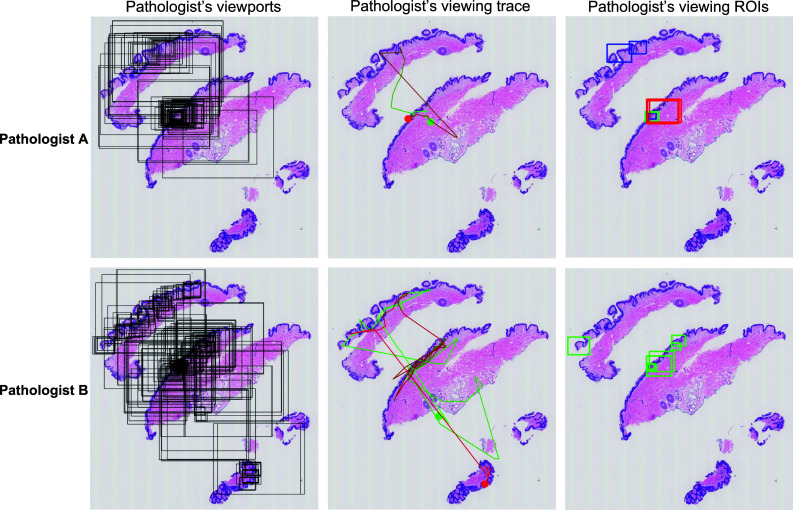
Each row visualizes a different pathologist’s viewing patterns and behaviors. Left: All viewports are shown in rectangular regions with black borders. Middle: Traces of the viewports by connecting the center of rectangles shown on the left, starting the viewing process from the green circle, and ending viewing of the case with the red circle. Right: Viewing ROIs extracted from all viewports on the left using zoom peaks (blue), slow pannings (red), and fixations (green)

In histopathologic diagnosis, the field of view holds significance for pathologists, as they can explore digital cases by zooming in and out. Lower magnification viewports encompass a larger area of the WSI. To maintain control over the size of extracted viewports using this methodology and to identify more precise regions within the images, we exclusively consider viewports with a magnification greater than 5 × . In the following sections, we refer to these regions as viewing ROIs. Note that these regions are not necessarily related to the final diagnosis given to a case by the expert and may include distracting regions as well as diagnostic regions. Figure 1 shows how viewing behaviors of different pathologists differ while viewing the same case which results in different viewing ROIs.

#### Generating Viewing Heatmaps

**Fig. 2:**
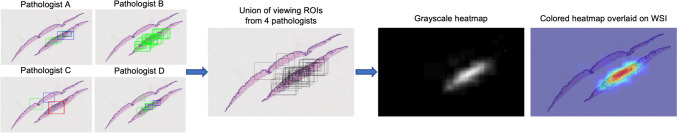
Left: Extracted viewports from four different pathologists (see the “Data Split” section for pathologists’ selection criteria) independently viewing the same case. Middle: Union of all the viewports shown on the left column. Right: Generated grayscale heatmap of the middle column viewports based on the viewports’ duration and the colored version overlaid on top of the WSI, highlighting the important regions

Each skin biopsy case in our dataset is independently viewed by an average of 5 community pathologists. We generated a single heatmap for each case by getting the union of all viewing ROIs extracted from pathologists’ interpretations as described in the “Pathologists’ Viewport Data” section and shown in Fig. 2. However, to reduce the distracting areas viewed by pathologists, we only consider interpretations with a maximum of 1-class diagnosis error as defined in the “Consensus Reference Diagnosis and Relationship to Diagnostic Accuracy” section. We define an accurate diagnosis as a diagnosis in agreement with the consensus reference classification and diagnosis error as a difference between the pathologist’s diagnosis and the consensus diagnosis.

We generated a pixel-level heatmap based on the duration each pixel was viewed. The total viewing time for each pixel across all viewports was accumulated to determine its heatmap value. These heatmaps were then normalized to values between 0 and 1. This means regions with a lower value (less bright regions) are of lower importance and pixels with higher values (brighter regions) are more important in the diagnosis as they have been viewed more during diagnosis. These heatmaps are used as the ground truth in this study.

#### Method and Experiment Setup

Autoencoders, as initially conceptualized [50], are designed to reconstruct their input. They are mainly composed of an encoder network that maps input data into a low-dimensional latent space and a decoder network that reconstructs the input from this latent space representation. The objective is to ensure the reconstructed version closely resembles the original. Encoder-decoder models are optimized by minimizing the disparity between the input and output images, typically by using mean squared error (MSE) as a loss function. This equips them with the proficiency to reconstruct images from condensed representations with high fidelity.

Deep learning has shown considerable potential in medical image analysis applications in recent years [51–59]. However, translating research breakthroughs into clinical tools remains a challenging process [60]. One of the primary barriers is the scarcity of high-quality labeled data required for developing accurate models [61]. Transfer learning [62] offers a solution by leveraging a model pre-trained on a different task, like ImageNet [63], as a foundation for a novel task. In the context of encoder-decoder architectures, transfer learning can be used to fine-tune a pre-trained model as the encoder to extract features for a new task.

**Fig. 3:**
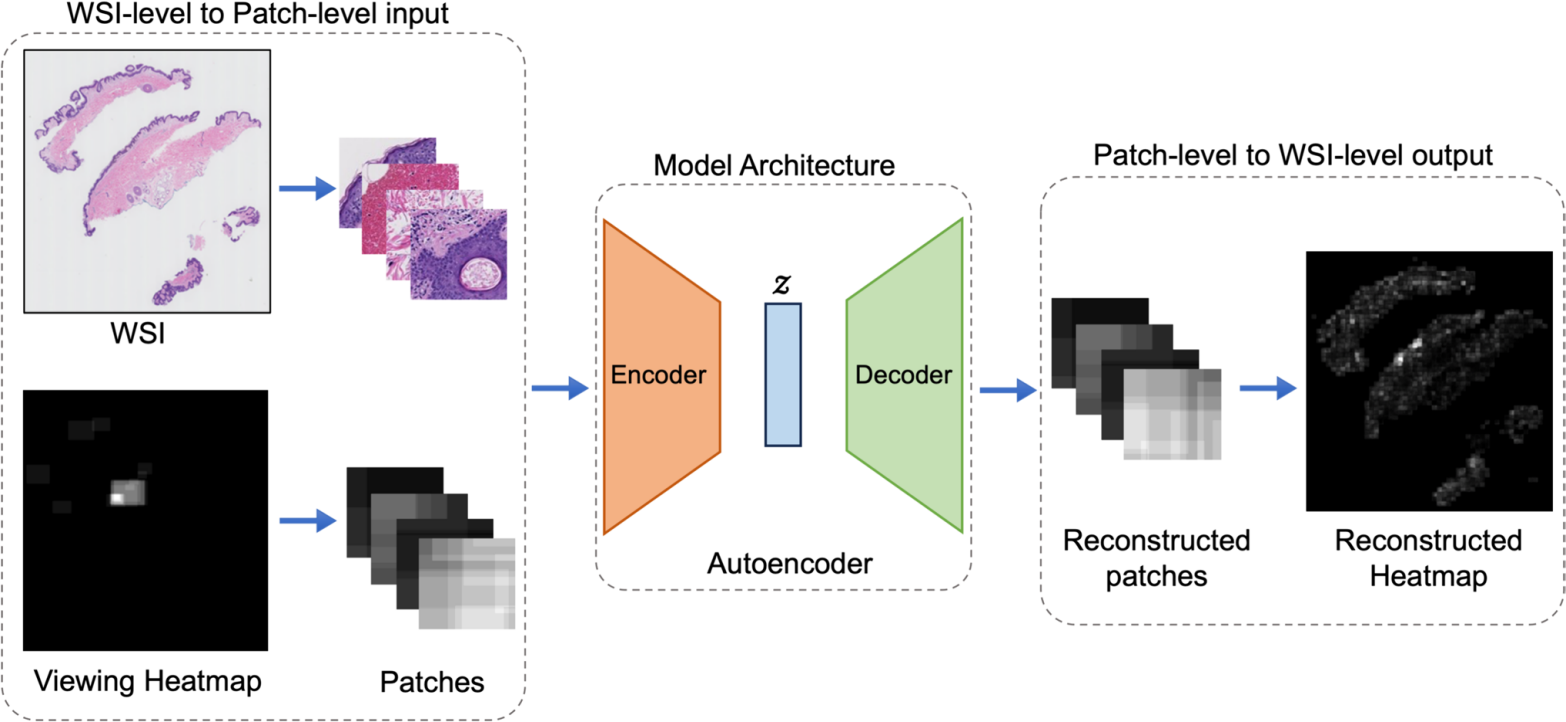
Pipeline of the ROI detection model. The encoder transforms input patches into a latent representation z, while the decoder then reconstructs these inputs from the latent space back into the original pixel space. See the “Method and Experiment Setup” section for details of the encoder and decoder architectures of the model

In this study, we used three model architectures to reconstruct input images as illustrated in Fig. 3: a convolutional autoencoder (ConvAE), a U-Net, and an Attention U-Net with attention.

- **ConvAE:** We initialized the encoder with the ResNet-18 [64] model pre-trained on ImageNet [63]. Our decoder consisted of 5 deconvolution layers with ReLU activation, except for the final layer, which used sigmoid activation.
- **U-Net and Attention U-Net:** We used the implementation of U-Net [65] by Yakubovskiy [66]. Both models were initialized with ResNet-34 [64] pre-trained on the ImageNet dataset as the encoder and a standard U-Net decoder. Figure 3 demonstrates the pipeline of our system. The Attention U-Net incorporated spatial Squeeze and channel Excitation (scSE) attention modules [67].

For our experiments, we used the Adam optimizer with a learning rate of 0.001. For the 256 × 256 patch size experiments, we used 2 GPUs with a 64 batch size. For the 512 × 512 patch size experiments, we used 4 GPUs with a 32 batch size. Models were trained on the training set and validated using the validation set to stop training when the model’s performance started to degrade and avoid overfitting. All experiments were done on NVIDIA GeForce GTX 1080 GPUs with 8 GB memory each.

For image pre-processing, we used the ImageNet standard normalization, setting the mean to (0.485, 0.456, 0.406) and the standard deviation to (0.229, 0.224, 0.225). Additionally, we employed a diverse set of image augmentations, including horizontal and vertical flips, random cropping, sharpening, embossing, brightness adjustments, hue and saturation modifications, grayscale conversion, and contrast adjustments. These augmentations were applied in a randomized sequence to enhance the robustness and variability of our dataset.

In addition to our approach, we also re-implemented the method by Mercan et al. [35]. Originally designed for ROI detection in breast biopsy images, we adapted, trained, and tested this model using our M-Path dataset. The method follows a bag-of-words approach [68] for feature construction. By using a sliding window, the WSI is divided into 1024 × 1024 bags, overlapping by 512 pixels in both dimensions. Each bag is further divided into 128 × 128 non-overlapping words (8 × 8 words per bag). Using the K-means clustering algorithm, words are grouped into 40 clusters based on their color (Lab) and texture (LBP) features extracted earlier. For each bag, a frequency histogram is calculated, representing the distribution of the 8 × 8 patches across the 40 clusters. Next, viewing ROIs are extracted as described in the “Extracting Viewing ROIs” section, and bags are labeled as either positive (ROI) or negative (non-ROI) based on their intersection with the extracted viewing ROIs. Finally, we employed a Random Forest classifier to distinguish between ROI and non-ROI. More details of this method can be found in [35].

### Evaluation

In this section, we introduce the methods we used for evaluating the performance of our model. First, we define the metrics used for the quantitative assessment of the model. Second, we explain the clinical evaluation of the study done by three dermatopathologists. Finally, we show how the proposed framework can enhance computer-aided diagnosis (CAD) systems.

#### Quantitative Assessment

To evaluate our results at an individual patient skin biopsy WSI level, we stitched patches generated by our model together to generate the WSI-level heatmap. We used mean squared error (MSE) and structural similarity index (SSIM) to measure the similarity between the reconstructed heatmaps and the ground truth. Additionally, we employed standard pixel-level segmentation metrics, including Intersection over Union (IoU), precision, recall, and F1-score to assess model’s performance. Collectively, these metrics offer a comprehensive assessment of the model’s capability.

- **MSE:** Measures the average squared differences between the predicted and actual values, commonly used to assess an autoencoder’s performance. In our context, the predicted value corresponds to the model-generated heatmap, while the actual value is the ground truth from pathologists’ viewing behavior. MSE is defined below in Eq. (1) where *m* and *n* are the dimensions of the image and *y*i,j and *ý*i,j are (i, j) pixel values at input and output images, respectively.
  1 $$MSE={\sum }_{i=0}^{m}{\sum }_{j=0}^{n}{\left({y}_{i,j}-{\acute{y}}_{i,j}\right)}^{2} /m *n$$
- **SSIM:** Measures the similarity between two images by comparing their structural information, including luminance, contrast, and structure. It provides a score ranging from 0 to 1, with 1 denoting identical images. In our study, we calculated the SSIM score between the model’s reconstructed heatmap and the ground truth heatmap. The SSIM score was used as an objective measure of the similarity between the two images, with a higher score indicating a better match. The formula for calculating SSIM is provided in Eq. (2) where *l*, *c*, and *s* represent the luminance, contrast, and structure components. The parameters *α*, *β*, and *γ* are used to weight each component, with typical values of 0.01, 0.03, and 0.03, respectively. and  are the input and output images, respectively.
  2 $$SSIM = (ly,\acute{y} )^{\upalpha}*c (y,\acute{y})^{\upbeta}*{s(y,\acute{y} )}^{{\upgamma}}$$
- **IoU, precision, recall, and F1-score:** Measure the overlap between the generated heatmap and the ground truth, revealing how much of the ground truth is identified by the model. First, we apply a binary thresholding for each heatmap with a threshold of 0.5, categorizing pixels with values above this threshold as “1” (ROI) and below as “0” (non-ROI). We conducted experiments with several threshold values—0.4, 0.45, 0.5, 0.6, and 0.7—and found 0.5 to be the best threshold for this task. Based on this binary thresholding, the definitions of true positive (TP), false positive (FP), and false negative (FN) are given below:
  -
    - TP refers to the number of pixels correctly predicted as ROI.
    - FP denotes the pixels incorrectly predicted as ROI.
    - FN represents the ROI pixels that were missed by the model.
  - 3 $$\begin{aligned} \text{Precision}& =\text{ TP}/(\text{TP}+\text{FP}) \\ \text{Recall}& =\text{ TP}/(\text{TP}+\text{FN}) \\ \text{F}1 & = 2*\text{Precision}*\text{Recall}/(\text{Precision}*\text{Recall}) \\ \text{IoU}& =\text{ TP}/(\text{TP}+\text{FP}+\text{FN}) \end{aligned}$$

#### Clinical Evaluation by Dermatopathologists

We asked three co-author dermatopathologists to review the model-generated heatmaps on the test set containing 35 WSIs and grade the model’s performance using discrete scoring. It’s crucial to note that these dermatopathologists are different from the community pathologists whose viewing behavior was used to train our model. Their task was to evaluate the segmentation of the whole slide images. Each dermatopathologist received an individual Google Forms survey. Each of the 35 WSIs was presented at 10 × magnification alongside the grayscale model-generated heatmaps. An overlay of the heatmap on the corresponding WSI was also available for better clarity. The dermatopathologists addressed two questions aimed at discerning whether the model was over-detecting or under-detecting essential regions:

1. Does the heatmap closely correlate with your viewing behavior? Rate yes, somewhat, or no.
2. Does the most intense region of the heatmap include the region most representative of your diagnostic impression? Rate yes or no.

It’s essential to underscore that human analysis, particularly within medical evaluations, embodies a degree of inherent subjectivity. Recognizing this, our dermatopathologists convened in a collaborative session before their individual case analyses to develop standardized definitions to follow for each of the two clinical questions. This meeting enabled them to arrive at a mutual understanding of the interpretation of the cases. This consensus-building initiative was strategically implemented to instill a level of uniformity in the evaluation process, aiming to reduce individual biases. We analyzed the feedback from all three surveys, considering each one individually and collectively. We categorized the responses for Q1 and Q2 into distinct labels. Specifically, for Q1, the responses were categorized as “No,” “Somewhat,” and “Yes.” For Q2, the responses were categorized as “No” and “Yes.” 

#### Computer-Aided Diagnosis

The proposed ROI detection framework generates heatmaps that can be used as supplementary signals to train Diagnostic model. We utilize the architecture presented in [69] for this purpose. In this architecture, multiple masks can be appended as additional channels to the input image. Using a MobileNetV2 backbone [70], we extract features from the images at three scales of 7.5x, 10x, and 12.5x. These feature vectors are subsequently fed into ScATNet [71] which aggregates information of the three scales to perform the diagnostic task using Transformer blocks. Specific details regarding the model architecture can be found in [69, 71]. We trained our models for 200 epochs on a single NVIDIA RTX A4000 GPU with 16 GB GPU memory. All the training details and hyperparameters are the same as those in [71].

We train two models for comparison: one using only WSIs and the other incorporating the heatmaps generated by our ROI detection model as a fourth channel added to the WSIs. We evaluate the models using F1-score (equation 3), as well as sensitivity (recall) and specificity as shown in equation 4. Given that this is a multi-class classification problem, TP, FP, FN , and TN are calculated by summing across all classes.

4 $$\begin{aligned}\mathrm{Sensitivity\ \left(recall\right.)}&=\mathrm{TP}/\left(\mathrm{TP}+\mathrm{FN}\right.)\\ \mathrm{Specificity}&=\mathrm{TN}/\left(\mathrm{TN}+\mathrm{FP}\right.)\end{aligned}$$

## Results

**Fig. 4:**
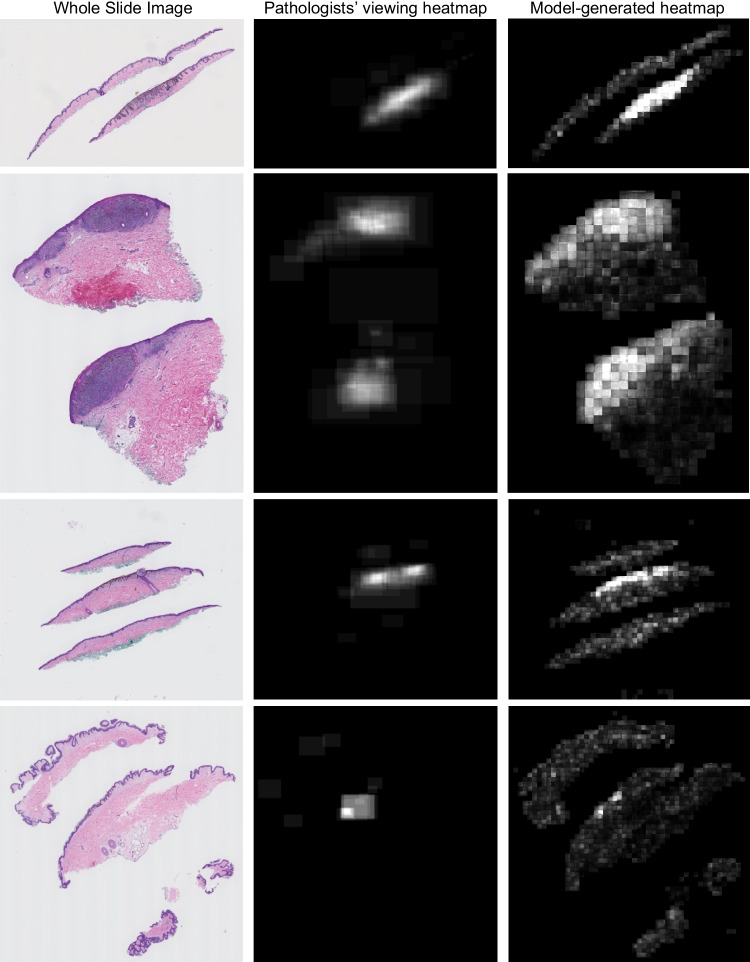
Visualized result for four example WSIs. Left: WSIs. Middle: Ground truth heatmaps from pathologists’ viewing ROIs (see the “Generating Viewing Heatmaps” section). Right: Model-generated heatmap on unseen data

In this section, we provide the results of our experiments. We present the results of our experiments and their improvement over the method by Mercan et al. [35] in Table 2. Experiments v1–v3 and experiments v4–v6 use patch sizes of 256 and 512, respectively. In order to validate the consistency of our model’s performance, we conducted multiple runs with three distinct random seeds and reported the average values for each metric. Our best model outperforms Mercan et al. [35] by 20% in precision, 11% in recall, 22% in F1-score, and 12% in Intersection over Union (IoU). Figure 4 shows heatmaps generated by our model on an unseen test set, alongside their ground truth viewing heatmaps. Additionally, we conducted experiments to investigate the effects of patch size and types of pathologists’ viewing behavior on the model’s performance. The results of these experiments are discussed in the subsequent sections.

**Table 2:** Results of experiments. All experiments are evaluated using the M-Path dataset (see the “Dataset and Pre-processing” section)

Model architecture	Patch size	Avg. MSE	Avg. SSIM	Precision	Recall	F1	IoU
v1: ConvAE	256	0.0146	0.876	**0.28**	0.49	0.36	0.18
v2: U-Net	256	0.0149	0.709	0.27	**0.53**	**0.36**	**0.20**
v3: Attention U-Net	256	0.0147	0.712	0.26	0.45	0.33	0.18
v4: ConvAE	512	0.0147	0.692	0.20	0.48	0.28	0.15
v5: U-Net	512	0.0155	0.682	0.19	0.44	0.27	0.14
v6: Attention U-Net	512	0.0157	0.677	0.18	0.53	0.27	0.15
Mercan et al. [35]	-	-	-	0.08	0.42	0.14	0.08

**Fig. 5:**
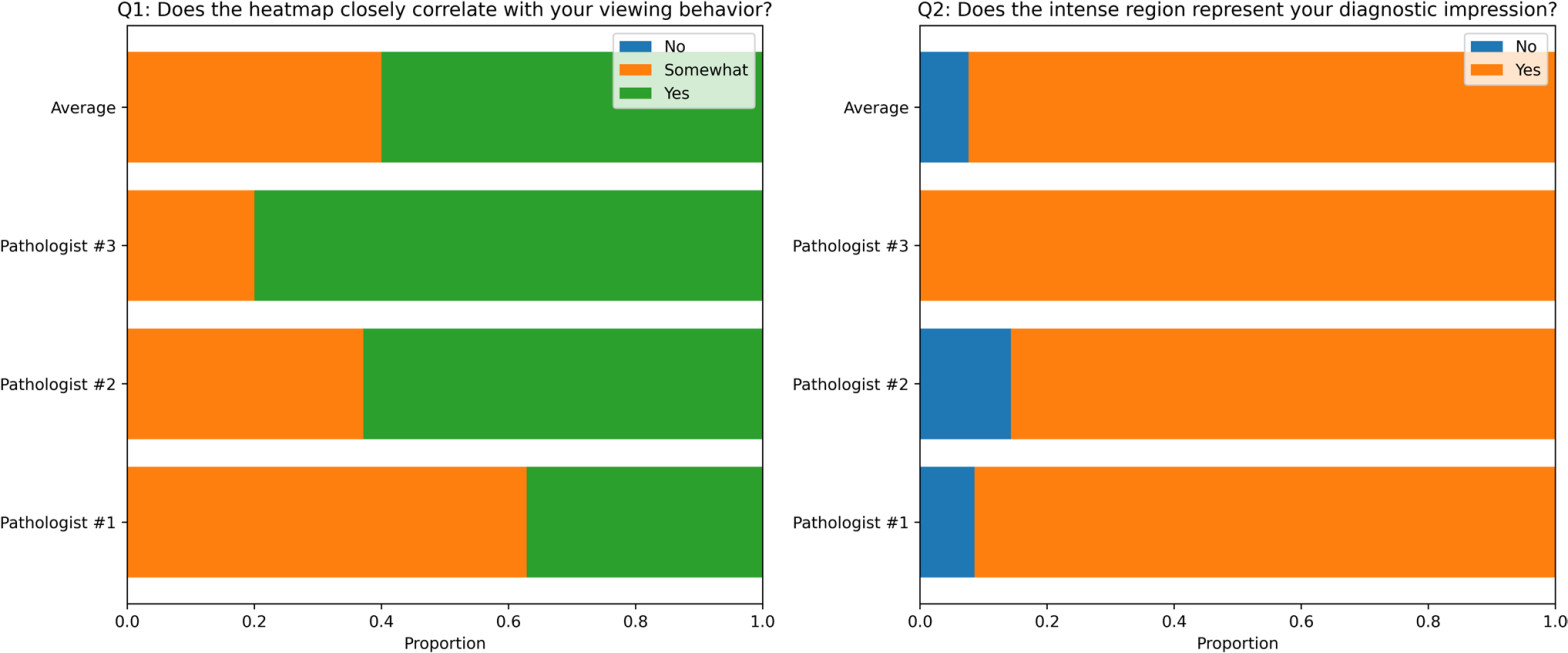
Proportion of responses from individual pathologists and the average of all three pathologists for **a** Q1: Does the heatmap closely correlate with your viewing behavior? and **b** Q2: Does the most intense region of the heatmap include the region most representative of your diagnostic impression? (See the “Clinical Evaluation by Dermatopathologists” section for the description of the clinical evaluation)

WSIs often contain multiple important regions. However, the ground truth heatmap, generated from pathologists’ viewing behavior (see the “Generating Viewing Heatmaps” section), might not encompass all of these important regions. We observed that our model identified certain areas with characteristics akin to these critical regions, leading to a high false-positive rate. Consequently, the conventional pixel-level segmentation metrics highlighted in the “Quantitative Assessment” section do not entirely reflect the model’s efficacy. To address this, we performed a clinical evaluation, involving three dermatopathologists (see the “Clinical Evaluation by Dermatopathologists” section). This evaluation comprised two questions, measuring the resemblances between the pathologists’ assessment of the critical regions of the WSI and the model-generated heatmaps. To provide a clear representation of the feedback, we used spineplots to display the proportion of responses within each category for each pathologist, as well as the average proportion across all pathologists. Figure 5 visualizes the distribution of responses, providing insights into the consensus among pathologists and highlighting any variations in their evaluations. The outcomes from this assessment demonstrate the capability of our model to generate a heatmap that replicates the regions that a pathologist would view and also to highlight the regions’ most representative of the final diagnosis.

**Fig. 6:**
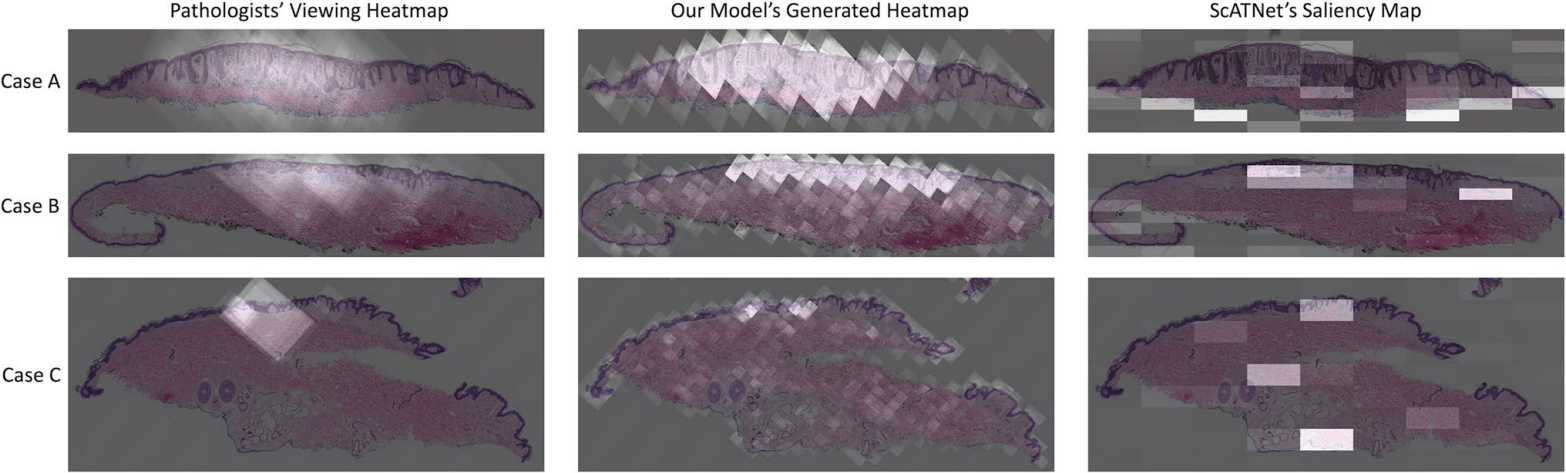
Comparison of the heatmaps generated by our ROI prediction model (middle) and the saliency maps of ScATNet [71] trained for diagnosis using WSIs (right). Ground truth heatmaps, based on pathologists' viewing behavior, are shown on the left

In Table 3, we present the results of our diagnostic experiments. Each model was trained using 5 different random seeds and we are reporting the average scores of each experiment. The results indicate an improvement in the diagnostic performance of the model when the heatmap is included as an additional channel in the input. Additionally, saliency analysis using gradients helps identify relevant areas in an input image that contributed to the prediction. We compare the heatmaps generated by our model with the saliency maps of the ScATNet [71] model trained only on WSIs. Figure 6 shows that our model’s heatmaps are more aligned with pathologists’ viewing heatmaps.

**Table 3:** Results of WSI diagnosis. All numbers are average scores over 5 random seeds per experiments

Model Input	Micro F1-score	Specificity	Sensitivity
WSI	0.59	0.86	0.59
**WSI + Heatmap **	**0.63**	**0.88**	**0.63**

### Patch Size

**Fig. 7:**
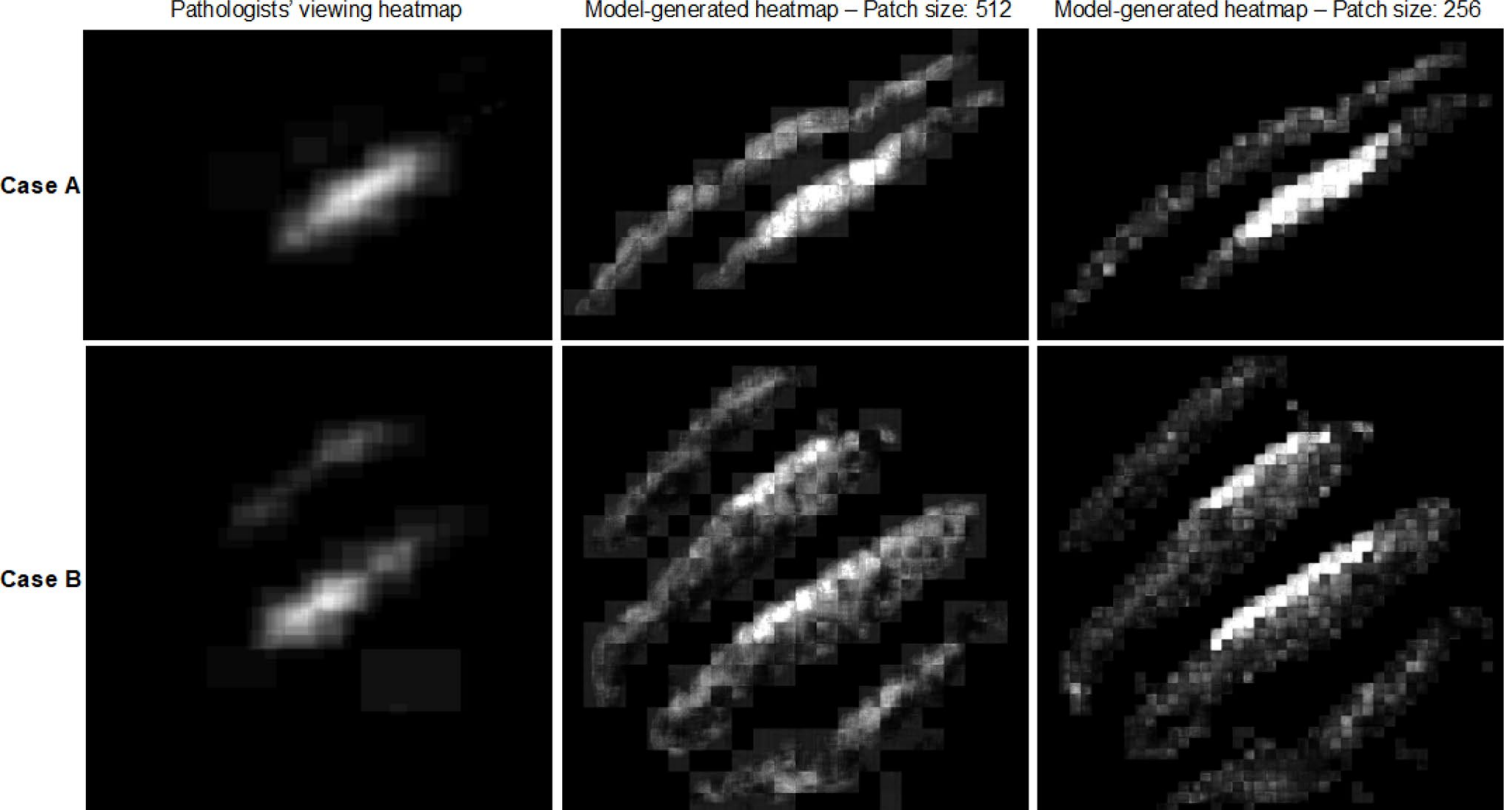
Left: Heatmap generated using pathologists’ viewing ROIs (see section the “Generating Viewing Heatmaps” section). Middle and right: Heatmaps generated by the model on unseen data with 512 × 512 and 256 × 256 patch sizes, respectively

To investigate the impact of patch size on the performance of our model, we set up our experiments with two different patch sizes: 256 × 256 and 512 × 512. A summary of the number of training and testing samples is provided in Table 1. By reducing the size of patches, the model loses insight into the location of these patches and their neighbor patches. On the other hand, increasing the size of patches requires more computing resources and higher training time. The results of these experiments show that a smaller patch size results in a more fine-grained heatmap, which is more similar to the original heatmaps. Figure 7 shows the results from using 256 × 256 patches and 512 × 512 patches compared to the ground truth heatmap.

### Consensus Reference Panel vs Community Pathologists’ Viewport Data

**Fig. 8:**
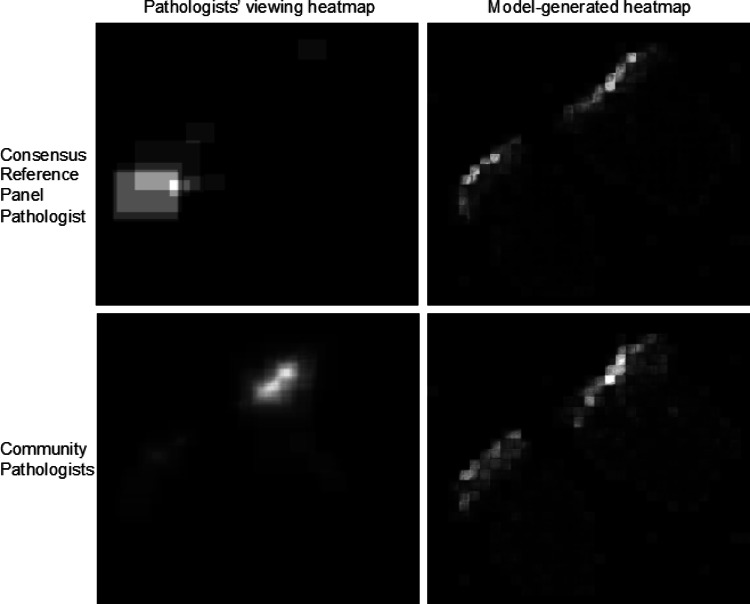
Top: The consensus reference panel pathologist ground truth heatmap and its model-generated heatmap. Bottom: Community pathologists ground truth heatmap and its model-generated heatmap

We investigated how viewing behavior from two groups of pathologists, community and M-Path consensus reference panel pathologists, would impact the performance of the model in detecting more precise ROIs. Hence, we used viewing behavior heatmaps generated from viewports of these two groups as input for training our model. Figure 8 shows a comparison of the consensus reference panel and community pathologists’ viewing behavior heatmaps and the corresponding results generated using these heatmaps during training. Heatmaps of the consensus reference panel are less cluttered and focused on a few smaller regions whereas community pathologists perform a more comprehensive scan of the slide.

## Discussion

Whole slide imaging has provided the opportunity to study the diagnostic viewing process of pathologists, yielding valuable insights that can be utilized to develop innovative training and evaluation programs as well as possibly using the data to improve computer-aided diagnosis systems. We have introduced an ROI detection system as the first step of the diagnosis process, aimed at assisting pathologists in quickly identifying relevant regions. The ROIs, identified using pathologists’ viewing behaviors such as zoom peaks, slow pannings, and fixations were utilized to generate a grayscale heatmap which guides our model to focus on crucial image regions. We employed three deep learning architectures for reconstructing the heatmaps. These regions may not necessarily represent the definitive ROIs of the digital slide but replicate a pathologist’s viewing patterns that can include distracting or misleading regions, providing a more realistic depiction of the diagnostic process.

Our model outperformed the Mercan et al. method [35], with an emphasis on high recall, capturing all relevant regions to reduce the chance of missing crucial information, despite potentially including some false positives. The use of viewport-extracted ROIs and square-shaped patches allowed our model to align closely with the ground truth in terms of shape and structure. In additional experiments, we analyzed the impact of patch size and type of pathologists’ viewing behavior on our model’s performance. Larger patch size had little effect on performance but required more computing resources. Models trained using the consensus reference panel pathologists’ viewing heatmaps produced fewer false-positive samples since these heatmaps highlight smaller image regions as these pathologists did not require a lot of scanning to find the ROIs. Consequently, the final output of the model generated from the viewing data of the consensus reference panel pathologists consisted of smaller and fewer ROIs.

The intrinsic complexity of ROI detection can lead the model to detect regions as ROIs that are not present in the ground truth set. However, this does not imply that these regions are insignificant. These regions can be ignored if found irrelevant by pathologists. The findings from our clinical evaluation demonstrate the effective performance of our model, despite its low precision. Moreover, the tracking software records visible regions in a rectangular shape, introducing unimportant surrounding regions and white space background, especially at lower zoom levels. Despite our efforts to minimize non-tissue patches during WSI pre-processing, the complete exclusion of unwanted regions was not achievable. Furthermore, the absence of eye tracking data restricts our ability to accurately determine the specific focus points of pathologists within these full viewports. Despite these limitations and challenges, our model demonstrated efficiency by simplifying and accelerating ROI annotation, thereby reducing costs.

We integrated the results of the ROI detection model into a computer-aided diagnosis system as supplementary signals and demonstrated that the performance of the diagnosis model improved with this addition. Moreover, we visualized the saliency maps of the diagnosis model trained solely on WSIs (without the heatmaps). Upon comparison, our model’s generated heatmaps showed greater alignment with pathologists’ viewing heatmaps than the saliency maps of the diagnosis model. 

In the field of ROI detection in histopathological images, our approach distinguishes itself by integrating pathologists’ viewing behavior data from their clinical review and interpretation of each case into the model’s training; this viewing behavior data is quite distinctive from the many methods that predominantly rely on manually labeled ROIs. While numerous studies have focused on an object detection approach, our analysis suggests that this might not be the optimal paradigm for such a nuanced task. ROIs in histopathological images differ from standard objects found in natural images, challenging exact bounding box comparisons. Instead, our model uses behavior-driven heatmaps to effectively highlight diagnostically relevant regions. This unique methodology, grounded in real-world clinical insights, positions our approach a notch above most state-of-the-art techniques, which often overlook the importance of replicating the intricate clinical viewing behavior of pathologists. Moreover, the lack of available public datasets that capture viewing behavior in histopathological images is a recognized challenge. This restricts external validation of our methodology on diverse datasets and poses a barrier to direct comparisons with other existing techniques.

As the future direction, the addition of precise eye tracking data would help determine the exact focus of pathologists within the full rectangular viewports, potentially refining the output of the model. The proposed ROI detection model can be used for developing automated diagnosis systems by locating crucial regions rather than processing the entire slide. Additionally, it would be beneficial to explore the optimal integration of these models into practical, clinical settings and understand how this technology can be more tailored to individual needs for pathologists at varying experience levels. This is because integrating CAD models into healthcare practice requires strict regulatory standards, exhaustive validation, and certification to ensure patient safety and compliance with medical protocols. Moreover, scalability is a pivotal concern, as models proven in controlled experimental settings must be adeptly tailored to accommodate the heterogeneity of data encountered in practical clinical environments. This type of algorithm to identify important image ROIs could be quite helpful as a resource for training and educating the next generation of pathologists.

## Conclusions

In this study, we explored the complex viewing behaviors of pathologists in diagnosing a slide, gaining insight into their decision-making process. This understanding has the potential to enhance the training and education of pathologists and to facilitate the development of computer-aided and AI tools for supporting pathology diagnosis. Achieving human-level performance in AI often necessitates a substantial volume of accurately labeled data, a significant challenge addressed by automated labeling methods. The new method described in our paper aims to mitigate the data labeling challenge by leveraging the combined expertise of human experts and algorithmic models. We utilized viewport-extracted ROIs, and our model achieved improved performance compared to previous methods. As pathology labs transition to digital modalities, the collection of viewing behavior data from pathologists can be scaled up. Integrating this amassed data with our proposed framework offers a faster and less expensive alternative to manual ROI annotation by pathologists. The validation results of our study show an increase of 20% in precision, 11% in recall, 22% in F1-score, and 12% in IoU compared to previous methods. We demonstrated how this ROI detection system can be integrated with a CAD system to im-prove its performance, further indicating that the predicted heatmaps are sufficiently accurate, making them valuable priors for guiding the focus of attention in future medical image analysis tasks. In conclusion, deep learning has revolutionized computer-aided diagnosis models by enabling the extraction of complex patterns directly from medical images. Our findings contribute to enhancing the accuracy and efficiency of CAD systems, supporting clinical practices, and fostering advancements in the field of digital pathology.

## Data Availability

Due to stringent privacy considerations, ethical constraints, and institutional policies, our dataset is not publicly available for general release. However, interested individuals can contact authors for more information. Our codebase is available at: https://github.com/fGhezloo/ROI-Localization-melanoma.
